# The Impact of Infection on Population Health: Results of the Ontario Burden of Infectious Diseases Study

**DOI:** 10.1371/journal.pone.0044103

**Published:** 2012-09-04

**Authors:** Jeffrey C. Kwong, Sujitha Ratnasingham, Michael A. Campitelli, Nick Daneman, Shelley L. Deeks, Douglas G. Manuel, Vanessa G. Allen, Ahmed M. Bayoumi, Aamir Fazil, David N. Fisman, Andrea S. Gershon, Effie Gournis, E. Jenny Heathcote, Frances B. Jamieson, Prabhat Jha, Kamran M. Khan, Shannon E. Majowicz, Tony Mazzulli, Allison J. McGeer, Matthew P. Muller, Abhishek Raut, Elizabeth Rea, Robert S. Remis, Rita Shahin, Alissa J. Wright, Brandon Zagorski, Natasha S. Crowcroft

**Affiliations:** 1 Institute for Clinical Evaluative Sciences, Toronto, Canada; 2 Public Health Ontario, Toronto, Canada; 3 Dalla Lana School of Public Health, University of Toronto, Toronto, Canada; 4 Department of Family and Community Medicine, University of Toronto, Toronto, Canada; 5 Department of Health Policy, Management, and Evaluation, University of Toronto, Toronto, Canada; 6 Department of Laboratory Medicine and Pathobiology, University of Toronto, Toronto, Canada; 7 Department of Medicine, University of Toronto, Toronto, Canada; 8 Centre for Global Health Research, St Michael's Hospital, Toronto, Canada; 9 Centre for Research on Inner City Health, The Keenan Research Centre, Li Ka Shing Knowledge Institute, St Michael's Hospital, Toronto, Canada; 10 Toronto Public Health, Toronto, Canada; 11 Ottawa Hospital Research Unit, Ottawa, Canada; 12 Department of Population Medicine, University of Guelph, Guelph, Canada; 13 Department of Health Studies and Gerontology, University of Waterloo, Waterloo, Canada; 14 Public Health Agency of Canada, Guelph, Canada; 15 Department of Medicine, University of British Columbia, Vancouver, Canada; Indiana University and Moi University, United States of America

## Abstract

**Background:**

Evidence-based priority setting is increasingly important for rationally distributing scarce health resources and for guiding future health research. We sought to quantify the contribution of a wide range of infectious diseases to the overall infectious disease burden in a high-income setting.

**Methodology/Principal Findings:**

We used health-adjusted life years (HALYs), a composite measure comprising premature mortality and reduced functioning due to disease, to estimate the burden of 51 infectious diseases and associated syndromes in Ontario using 2005–2007 data. Deaths were estimated from vital statistics data and disease incidence was estimated from reportable disease, healthcare utilization, and cancer registry data, supplemented by local modeling studies and national and international epidemiologic studies. The 51 infectious agents and associated syndromes accounted for 729 lost HALYs, 44.2 deaths, and 58,987 incident cases per 100,000 population annually. The most burdensome infectious agents were: hepatitis C virus, *Streptococcus pneumoniae*, *Escherichia coli*, human papillomavirus, hepatitis B virus, human immunodeficiency virus, *Staphylococcus aureus*, influenza virus, *Clostridium difficile*, and rhinovirus. The top five, ten, and 20 pathogens accounted for 46%, 67%, and 75% of the total infectious disease burden, respectively. Marked sex-specific differences in disease burden were observed for some pathogens. The main limitations of this study were the exclusion of certain infectious diseases due to data availability issues, not considering the impact of co-infections and co-morbidity, and the inability to assess the burden of milder infections that do not result in healthcare utilization.

**Conclusions/Significance:**

Infectious diseases continue to cause a substantial health burden in high-income settings such as Ontario. Most of this burden is attributable to a relatively small number of infectious agents, for which many effective interventions have been previously identified. Therefore, these findings should be used to guide public health policy, planning, and research.

## Introduction

Evidence-based priority setting is increasingly important for rationally distributing scarce health resources and for guiding future health research. [1] Using different methodologies and measures, numerous studies have estimated population-level disease burden. [2]–[6] Although infectious diseases have regained prominence in high-income settings with recent outbreaks of emerging and re-emerging diseases such as severe acute respiratory syndrome (SARS), measles, and pandemic influenza, [7]–[9] a comprehensive assessment focusing on infectious diseases has not been conducted. We conducted the Ontario Burden of Infectious Disease Study (ONBOIDS) to provide a detailed comparison of a wide range of infectious diseases, to inform planning and decision-making, and to establish a baseline for future evaluations of public health interventions.

## Methods

### Ethics Statement

Ethics approval was obtained from the Research Ethics Board of Sunnybrook Health Sciences Centre, Toronto, Canada. This study used routinely collected health information from the province of Ontario and did not require informed consent from participants. Vital statistics and reportable infectious disease data were provided to the study researchers in the form of aggregate counts of deaths/infections by specific age-sex strata. The hospitalization and physician services data used contained no personal identifiers. The use of either aggregate data or data without personal identifiers precluded the need to obtain informed consent. Additionally, the Institute of Clinical Evaluative Sciences (ICES) is named as a prescribed entity under section 45 of the *Personal Health Information Protection Act* (Ontario Regulation 329/04, Section 18). Under this designation, ICES can receive and use health information without consent for purposes of analysis and compiling statistical information about the health care system of Ontario.

### Study design

The methodology for ONBOIDS was adapted from the Global Burden of Disease (GBD) and the Population Health Impact of Disease in Canada studies. [1], [10] These studies use composite measures combining mortality and morbidity to assess population health against a pre-specified ideal. Mortality and incidence data are compiled from various sources to estimate the future burden associated with incident cases of disease over a one-year period.

We included 51 infectious agents causing disease associated with one or more of the following characteristics: 1) severe morbidity/mortality; 2) high incidence; 3) historically relevant (i.e., legally reportable to public health authorities in Ontario); and 4) of emerging interest (e.g., new prevention program, extensive media attention). Some important pathogens were not included because data necessary to reliably assess incidence, mortality, and/or morbidity were not available in Ontario (e.g., norovirus, rotavirus, *H. pylori*). We also assessed the burden of 16 syndromes, defined as non-specific conditions that may be caused by different pathogens.

### Study population and setting

Ontario is Canada's most populous province with 12.2 million residents as of 2006, of whom 14% were 65 years or older, 85% lived in urban areas, 2% were Aboriginal, and 28% were born outside of Canada. [11] The top five countries of origin for immigrants were China (including Hong Kong Special Administrative Region), the United Kingdom, India, Italy and the Philippines. [12] In 2006, Ontario was estimated to have approximately 39,000 individuals who had ever injected drugs. [13] All provincial residents have free access to hospital care and essential physician services through universal health insurance.

### Outcome measure

We quantified the burden for each disease using the health-adjusted life year (HALY). The HALY is a composite measure of the gap between ideal and actual health that incorporates both mortality and morbidity. The HALY includes both death occurring before a pre-specified maximal life expectancy (i.e., years of life lost due to premature mortality [YLL]) and years of healthy life lost due to suboptimal states of health associated with disease (i.e., year-equivalents of reduced functioning due to disease [YERF]). [10]

YLL are computed by multiplying age- and sex-specific counts of deaths due to a particular cause by the remaining life expectancy for that age and sex stratum and then summing across the age-sex strata to obtain the YLL for each infectious disease.

Each infectious disease had one or more health states. For example, the health states for human immunodeficiency virus (HIV) were HIV, acquired immunodeficiency syndrome (AIDS), and terminal AIDS. To calculate YERF, we multiplied the age- and sex-specific number of incident cases of each associated health state by the average duration of the health state and the severity weight derived for that health state. We summed the YERF, first for each age-sex group and then across health states to obtain the YERF for each disease. Details are provided in the Supporting Information (Text S1).

HALYs are considered an umbrella term for the more familiar quality-adjusted life years (QALYs) used in health economics, and disability-adjusted life years (DALYs) used in the GBD study. [1], [14] The calculations for HALYs and DALYs are computationally similar, but our study differed from the GBD methodology in several respects: 1) the GBD methodology utilizes a standard life expectancy table (Coale and Demeny West level 26 model life table) [15] when calculating YLL, whereas we used Ontario life tables for 2001; 2) with the GBD methodology, disability weights are generated by expert opinion, while we derived severity weights for health conditions using the classification and measurement system of functional health methodology, [16] which yields a more comprehensive and internally consistent set of preference weights for both established and novel health states; 3) the GBD DALYs generally incorporate age-weighting (i.e., more weight is given to years lost in young adulthood), whereas we used uniform age-weights (i.e., no increased weighting for any age groups) since the ethical basis as well as the calculation of age weights is highly contested; [17], [18] and 4) the GBD methodology discounts future life years at a rate of 3% (i.e., future life years are assigned less value than those lived today), whereas we did not discount life years since discounting health effects is controversial and there is no consensus on the appropriate discount rate. [19], [20] We chose to use the more generic HALY.

### Data sources

For each disease/organism, we calculated three-year averages for incidence and mortality using the most recent available data.

**Mortality:** Recent mortality data were used as a proxy for future mortality resulting from present incident cases. Mortality data for 2005–2007 using the Tenth Revision of the International Classification of Diseases (ICD-10) were collected from medically-certified death certificates by the Ontario Office of the Registrar General.

**Incidence:** We sought to identify all incident cases occurring during 2005–2007 using data from several sources. We obtained counts of reportable infectious diseases from Ontario's integrated Public Health Information System (iPHIS). Where possible, we adjusted for under-diagnosis and under-reporting using estimates from epidemiologic studies from comparable populations. We also used epidemiologic studies and expert opinion to determine the distribution of the health states typically experienced by new cases. For diseases where reported cases do not reflect incident cases due to delays from time of infection to symptomatic presentation, diagnosis, and reporting (HIV, hepatitis B virus [HBV], and hepatitis C virus [HCV]), we used mathematical models to generate more accurate estimates of disease incidence and resulting health states. [21], [22]

Healthcare utilization data were used to estimate the incidence of infectious diseases where reportable disease data would be suboptimal, including non-reportable pathogens, pathogens for which only a subset of infections are reportable (e.g., invasive *Streptococcus pneumoniae*), and syndromes for which etiologic agents are not usually defined by laboratory testing. These data were collected from validated linked population-based databases; data on physician visits, emergency department visits, and hospitalizations were abstracted from the Ontario Health Insurance Plan physician billing claims database, the National Ambulatory Care Reporting System, and the Canadian Institute of Health Information's Discharge Abstract and Same-Day Surgery databases, respectively. [23]–[28] Encrypted health card numbers were used as unique identifiers for linkage of individuals across datasets. Generally, the healthcare utilization data were used to determine episodes of syndromes that can be caused by multiple infectious agents (e.g., pneumonia). These cases were attributed to various pathogens based on estimates from epidemiologic studies. Repeat healthcare utilization events for the same patient were considered to represent a single episode of infection if they occurred within a pre-specified time period (Table S1).

The Ontario Cancer Registry (OCR), which records all cases of cancer except for non-melanoma skin cancer, was used to obtain data on the incidence of cancerous health states for selected infectious diseases (hepatocellular cancer for HBV and HCV, and anogenital and oropharyngeal cancers for human papillomavirus [HPV]). [29]

### Disease models

To illustrate the four main approaches used to estimate disease incidence and mortality, we present the following example pathogens. Full descriptions of the models used to estimate disease burden are available in the technical report at: http://www.ices.on.ca/file/ONBOIDS_FullReport_intra.pdf.

**Salmonella: using reportable disease data, adjusted for under-diagnosis and under-reporting:** To estimate disease incidence, we extracted reported cases of salmonellosis from iPHIS and applied a multiplier of 13 to adjust for under-diagnosis and under-reporting. [30] We used epidemiologic studies to determine the percentages of *Salmonella* cases that experienced the following health states: gastroenteritis – mild (did not seek medical care), gastroenteritis – moderate (saw a physician), gastroenteritis – severe (hospitalized), and septicaemia. The same denominator (i.e., adjusted number of cases) was used for all health states, but we assumed that individuals who had severe gastroenteritis or septicaemia had a prior episode of moderate gastroenteritis (i.e., saw a physician). We also used epidemiologic studies to determine the durations of the health states. These parameters are presented in Table S2. For mortality, we used vital statistics data to identify deaths coded as salmonellosis.

**HIV: using mathematical models:** We used the results of a mathematical model to estimate HIV incidence. We extracted HIV serodiagnoses among different exposure categories (men who have sex with men, injection drug use, mother-to-child transmission, blood product/transfusion recipient, emigrated from endemic area, heterosexual transmission) from the Ontario Central Public Health Laboratory. Results from detuned assays were used to distinguish between recent and remote HIV infection, and data from various studies were used to adjust for selection biases associated with HIV testing patterns among individuals at varying risks of HIV and HIV incidence among repeat testers, as well as determine the exposure category for those with missing risk factor information. [21] This model also produced estimates of AIDS incidence by using reported AIDS cases from the Ontario AIDS Surveillance Program and adjusting for reporting delays and under-reporting. [21] A simplifying assumption was that all cases of AIDS would reach the terminal phase of AIDS, although it is possible that some individuals with AIDS may die from other causes before the terminal phase. We determined durations of HIV and AIDS using modeled survival times. These parameters are presented in Table S3. We extracted HIV-coded deaths from vital statistics data to determine the number of deaths due to HIV.

**HPV: using cancer registry data and epidemiologic studies:** We estimated the incidence of, and mortality from, HPV-related cancers (i.e., cancers of the cervix, vulva, vagina, anal canal, penis, and oropharynx) using OCR data and vital statistics data, respectively, and we applied epidemiologic studies [31], [32] to determine the percentages of particular cancers attributable to HPV. We estimated the incidence of anogenital warts from an epidemiologic study. [33]

We adapted the approach developed by Statistics Canada for estimating the burden of cancers. [34] Briefly, individuals with cancer experience some or all of the following health states: diagnosis (good, fair, or poor prognosis), treatment (surgery, radiation, chemotherapy, and certain combinations of those options), remission (after one or more treatment modalities), and if death occurs within five years, palliative care and terminal care (last month of life) attributable to the cancer. We obtained stage distributions at diagnosis (i.e., proportion of individuals diagnosed at a particular stage) from the OCR for 2007 and 2008. Five-year relative survival estimates and treatment distributions were determined from epidemiologic studies and expert opinion. We made several simplifying assumptions. The first was that all incident cases underwent some form of treatment. In reality, some patients are too frail at diagnosis to receive treatment, but the proportion not undergoing any treatment is generally small. Second, we simplified the treatment options by not distinguishing between curative and palliative radiation therapy and by not considering chemotherapy associated with mild, moderate, and severe toxicity (keeping the mild form only). Third, we assumed that only deaths that occurred within five years of diagnosis were attributable to the cancer (i.e., those who survived past five years due to another diagnosis), and those who survived past five years had a life expectancy that was the same as the rest of their age-sex stratum. Fourth, we assumed that the proportion dying within five years was independent of the type(s) of treatment and that those who died experienced on average 2.25 years in remission (five years minus the time spent in palliative (five months) and terminal care (one month) divided by two = 4.5 years/2 = 2.25 years). Fifth, we assumed the reduction in functioning to be similar when in remission, regardless of the treatment modality (or modalities) employed. Finally, we did not consider recurrent cancers. These parameters are presented in Tables S4 and S5.

**S. pneumoniae: using healthcare utilization data and infectious disease syndromes:** We used healthcare utilization data and vital statistics to determine the number of episodes and deaths, respectively, of each of the syndromes resulting from *S. pneumoniae* infection (bacterial meningitis, septicaemia, pneumonia, septic arthritis, acute bronchitis, otitis media, and conjunctivitis). We also considered seizures, motor deficits, and deafness as sequelae of bacterial meningitis. We used epidemiologic studies to determine the percentage of each syndrome attributable to *S. pneumoniae* (to estimate both deaths and incident cases) and the duration of each health state. These parameters are presented in Table S6.

### Analysis

The calculation of HALYs was conducted using Microsoft Excel 2003 worksheet templates (available at http://www.who.int/healthinfo/global_burden_disease/tools_national/en/index.html). We conducted a sensitivity analysis estimating the burden of the top 20 pathogens using the GBD methodology (GBD standard life expectancy, disability weights, age-weighting, and discounting at a rate of 3%).

## Results

A total of 88,956 HALYs (729 per 100,000 population) were estimated to have been lost annually due to the 51 infectious agents and associated syndromes studied; 74,297 (83.5%) years of life were lost due to premature mortality (YLL) and 14,668 (16.5%) were due to YERF (table 1). There was modest correlation between YLL and YERF (Pearson correlation coefficient = 0.56). The ten highest burden pathogens were HCV, *S. pneumoniae*, *Escherichia coli*, HPV, HBV, HIV, *Staphylococcus aureus*, influenza virus, *Clostridium difficile*, and rhinovirus. YLL exceeded YERF for most pathogens. Nearly 50% of the burden was attributed to five pathogens. The top ten pathogens accounted for approximately 67% of total HALYs and the top 20 pathogens accounted for 75%.

**Table 1:** Number and proportion of average annual HALYs by infectious disease or pathogen, 2005–07, Ontario, Canada.

Rank	Infectious Disease/Pathogen	YLL	YERF	HALY	% of total HALYs
1	Hepatitis C virus	8823	983	9807	11.02%
2	*Streptococcus pneumoniae*	6669	1601	8270	9.30%
3	*Escherichia coli*	7485	341	7826	8.80%
4	Human papillomavirus	6191	1418	7609	8.55%
5	Hepatitis B virus	6918	86	7004	7.87%
6	Human immunodeficiency virus	5036	1312	6349	7.14%
7	*Staphylococcus aureus*	3740	400	4140	4.65%
8	Influenza virus	2482	1076	3558	4.00%
9	*Clostridium difficile*	3216	107	3323	3.74%
10	Rhinoviruses	125	1615	1740	1.96%
11	Respiratory syncytial virus	968	397	1364	1.53%
12	Parainfluenza virus	601	259	861	0.97%
13	Group A streptococcus	598	216	814	0.92%
14	*Haemophilus influenzae*	649	125	774	0.87%
15	Group B streptococcus	603	123	725	0.82%
16	Tuberculosis	675	16	691	0.78%
17	Legionellosis	588	40	628	0.71%
18	Chlamydia	19	442	461	0.52%
19	Varicella	150	303	453	0.51%
20	Adenovirus	301	150	451	0.51%
21	Coronaviruses	28	369	397	0.45%
22	Gonorrhea	17	371	388	0.44%
23	*Neisseria meningitidis*	177	152	329	0.37%
24	Herpes simplex virus	117	138	256	0.29%
25	Pertussis	0	220	220	0.25%
26	*Candida spp.*	126	92	218	0.24%
27	*Campylobacter spp.*	26	144	170	0.19%
28	*Blastomyces*	135	2	137	0.15%
29	*Pneumocystis jiroveci*	103	2	105	0.12%
30	Poliomyelitis	102	0	101	0.11%
31	*Aspergillus*	70	31	100	0.11%
32	*Salmonella spp. (excluding typhi/paratyphi)*	40	42	81	0.09%
33	West Nile virus	45	16	61	0.07%
34	*Giardia lamblia*	0	52	52	0.06%
35	*Listeria monocytogenes*	36	1	36	0.04%
36	*E. coli* O157	14	17	31	0.03%
37	Syphilis	13	18	31	0.03%
38	Hepatitis A	4	22	26	0.03%
39	Malaria	6	0	6	0.01%
40	*Histoplasma*	0	6	6	0.01%
41	*Shigella*	0	4	4	0.00%
42	Rubella virus	0	2	2	0.00%
43	*Yersinia enterocolitica* (not *Yersinia pestis*)	0	1	1	0.00%
44	Typhoid/Paratyphoid fever	0	1	1	0.00%
45	*Cryptosporidium*	0	1	0	0.00%
46	*Cyclospora cayetensis*	0	0	0	0.00%
47	Dengue	0	0	0	0.00%
48	Tetanus	0	0	0	0.00%
49	Mumps virus	0	0	0	0.00%
50	Measles virus	0	0	0	0.00%
51	Diphtheria	0	0	0	0.00%
	Syndromes due to other pathogens	17401	1957	19348	21.75%
	**Top 5 pathogens**	36087	4429	40516	45.55%
	**Top 10 pathogens**	50686	8940	59626	67.03%
	**Top 20 pathogens**	55837	11010	66848	75.15%
	**Total**	74297	14668	88956	100.0%

YLL = years of life lost to premature mortality; YERF = year-equivalents of reduced functioning; HALY = health-adjusted life years.

We estimated that these infectious diseases accounted for 5390 deaths (44.2 per 100,000) and 7,196,349 incident cases (58,987 per 100,000) annually in Ontario (table 2). *E. coli*, *S. pneumoniae*, HCV, HBV, and *C. difficile* accounted for the greatest numbers of deaths, while rhinovirus, influenza virus, *S. pneumoniae*, coronavirus, and *E. coli* accounted for the greatest number of incident cases.

**Table 2:** Number and proportion of average annual estimated deaths and incident cases by infectious disease or pathogen, 2005–07, Ontario, Canada.

	Mortality			Incidence		
Rank	Infectious Disease/Pathogen	Number of deaths	% of total deaths	Infectious Disease/Pathogen	Number of reported cases or healthcare utilization episodes	% of total cases/episodes
1	*Escherichia coli*	721	13.37%	Rhinoviruses	1615561	22.45%
2	*Streptococcus pneumonia*	634	11.77%	Influenza virus	621151	8.63%
3	Hepatitis C virus	405	7.52%	*Streptococcus pneumoniae*	518703	7.21%
4	Hepatitis B virus	367	6.81%	Coronaviruses	461767	6.42%
5	*Clostridium difficile*	327	6.07%	*Escherichia coli*	451268	6.27%
6	*Staphylococcus aureus*	294	5.46%	Respiratory syncytial virus	341471	4.75%
7	Human papillomavirus	263	4.87%	Parainfluenza virus	253292	3.52%
8	Influenza virus	256	4.75%	Adenovirus	203393	2.83%
9	Human immunodeficiency virus	141	2.62%	*Staphylococcus aureus*	158443	2.20%
10	Respiratory syncytial virus	96	1.79%	Group A streptococcus	118989	1.65%
11	*Haemophilus influenza*	62	1.16%	Varicella zoster virus	116049	1.61%
12	Parainfluenza virus	60	1.11%	*Haemophilus influenzae*	105076	1.46%
13	Legionellosis	59	1.09%	*Campylobacter spp.*	88566	1.23%
14	Group A streptococcus	49	0.91%	*Candida spp.*	71616	1.00%
15	Tuberculosis	46	0.85%	Chlamydia	61761	0.86%
16	Adenovirus	30	0.55%	*Salmonella spp.*	34693	0.48%
17	Group B streptococcus	18	0.33%	Gonorrhea	32234	0.45%
18	Varicella	11	0.20%	Human papillomavirus	15756	0.22%
19	Rhinoviruses	9	0.17%	Herpes simplex virus	14677	0.20%
20	*Candida spp.*	7	0.14%	Pertussis	8874	0.12%
	All other pathogens	1534	28.47%	All other pathogens	1903009	26.44%
	**Top 5 pathogens**	2454	45.54%	**Top 5 pathogens**	3668450	50.98%
	**Top 10 pathogens**	3505	65.03%	**Top 10 pathogens**	4744038	65.92%
	**Total**	5390	100.0%	**Total**	7196349	100.0%

**Figure 1:**
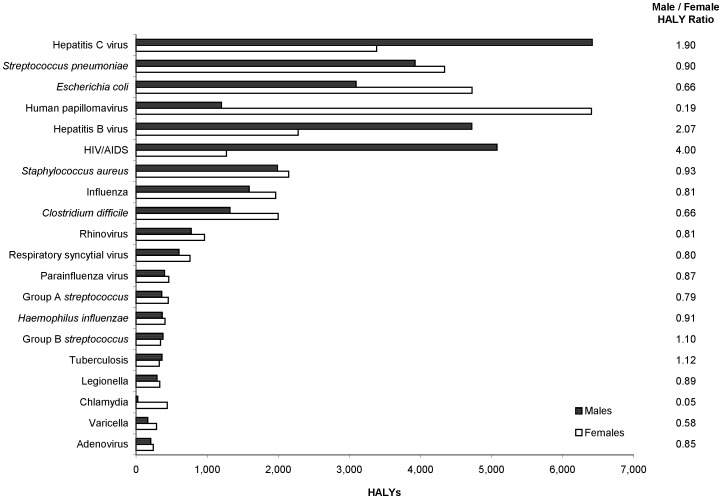
HALYs for the top 20 ranked infectious diseases or pathogens by sex, 2005–07, Ontario, Canada. For each infectious disease/pathogen, the dark-shaded bars represent the number of HALYs for males and the light-shaded bars represent the number of HALYs for females. The column to the right of the chart displays the male to female ratio of HALYs for each infectious disease/pathogen.

Among the 20 leading pathogens, the overall burden was comparable between the sexes. However, we observed a number of sex-specific differences (figure 1); HCV, HBV, and HIV had a greater impact on males, while HPV, *E. coli*, gonorrhea, and chlamydia had a greater impact on females.

**Figure 2:**
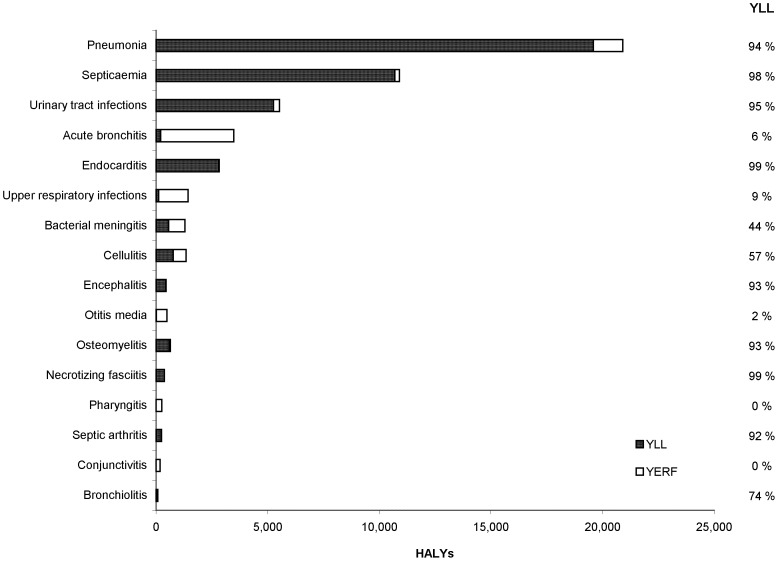
YLL, YERF and HALYs by infectious syndrome, 2005–07, Ontario, Canada. For each infectious disease syndrome, the dark-shaded portion of each bar represents YLL (years of life lost to premature mortality) and the light-shaded portion of each bar represents YERF (years equivalents of healthy life lost due to reduced functioning). The column to the right of the chart displays the percentage of HALYs that are due to YLL for each syndrome.

The top three selected infectious disease syndromes (pneumonia, septicaemia, and urinary tract infections) accounted for 74% of the total syndrome HALYs lost. Among these syndromes, pneumonia accounted for the greatest proportion of total HALYs (figure 2). For most syndromes, YLL accounted for a greater burden than YERF. The exceptions were acute bronchitis, upper respiratory tract infection, otitis media, pharyngitis, and conjunctivitis.

The ranking of infectious diseases using the GBD methodology was generally similar (Spearman rank correlation coefficient = 0.88), but the GBD methodology indicated a greater proportion of the burden attributable to premature morbidity (51.2%) versus mortality (48.8%; table 3).

**Table 3:** Burden of disease ranking of the top 20 infectious diseases or pathogens using Ontario Burden of Infectious Disease Study (ONBOIDS) and Global Burden of Disease (GBD) study methodologies, Ontario, Canada.

Rank	ONBOIDS methodology				GBD methodology				Change in Rank^a^
	Infectious Disease/Pathogen	YLL	YERF	HALYs	Infectious Disease/Pathogen	YLL	YLD	DALYs	
1	Hepatitis C virus	8823	983	9807	Human immunodeficiency virus	2712	6731	9443	+5
2	*Streptococcus pneumonia*	6669	1601	8270	Hepatitis C virus	4203	2042	6245	−1
3	*Escherichia coli*	7485	341	7826	*Streptococcus pneumoniae*	2617	3309	5926	−1
4	Human papillomavirus	6191	1418	7609	Human papillomavirus	2861	1704	4565	0
5	Hepatitis B virus	6918	86	7004	*Escherichia coli*	2833	550	3383	−2
6	Human immunodeficiency virus	5036	1312	6349	Hepatitis B virus	3177	143	3320	−1
7	*Staphylococcus aureus*	3740	400	4140	Influenza virus	948	1678	2626	+1
8	Influenza virus	2482	1076	3558	*Staphylococcus aureus*	1529	789	2318	−1
9	*Clostridium difficile*	3216	107	3323	Rhinoviruses	51	2047	2098	+1
10	Rhinoviruses	125	1615	1740	*Clostridium difficile*	1162	169	1331	−1
11	Respiratory syncytial virus	968	397	1364	Respiratory syncytial virus	363	547	910	0
12	Parainfluenza virus	601	259	861	Chlamydia	8	883	891	+6
13	Group A streptococcus	598	216	814	Adenovirus	117	676	793	+7
14	*Haemophilus influenzae*	649	125	774	Group A streptococcus	242	410	652	−1
15	Group B streptococcus	603	123	725	Parainfluenza virus	233	376	609	−3
16	Tuberculosis	675	16	691	Group B streptococcus	251	270	521	−1
17	Legionellosis	588	40	628	*Haemophilus influenzae*	254	241	495	−3
18	Chlamydia	19	442	461	Varicella	63	291	354	+1
19	Varicella	150	303	453	Tuberculosis	291	42	333	−3
20	Adenovirus	301	150	451	Legionellosis	228	94	322	−3
	**Total**	55837	11010	66848	**Total**	24143	22992	47135	

a – denotes the change in rank of an infectious disease/pathogen from its rank using the ONBOIDS methodology to its rank using the GBD methodology.

YLL = years of life lost to premature mortality; YERF = year-equivalents of reduced functioning; HALY = health-adjusted life years; YLD = years of life lived with disability; DALY = disability-adjusted life years.

## Discussion

We ranked the burden of disease associated with infectious pathogens and syndromes in the Canadian province of Ontario, determined that most of the burden was from premature mortality rather than morbidity, observed a number of sex-specific differences, and quantified the burden of syndromes caused by multiple pathogens. Some noteworthy themes among the top ten include pathogens with oncogenic potential (HCV, HBV, HPV), healthcare-associated infections (e.g., *C. difficile*, *S. aureus*), microorganisms present in the normal human microbiologic flora (e.g., *E. coli*, *S. aureus*), and diseases that are preventable by vaccines (HPV, HBV, *S. pneumoniae*, influenza virus). Notably absent among the top pathogens are those that have been successfully prevented through routine childhood vaccination (e.g. measles), a testament to the success of such programs.

Our annual estimated burden associated with infectious disease of 729 HALYs per 100,000 population is close to one-quarter of the 3017 HALYs per 100,000 population estimated for all cancers combined in a Canadian study using similar methodology. [35] This suggests that infectious diseases still contribute substantially to morbidity and mortality in high-income settings. One caveat for this comparison is that the burden of HPV-related cancers and hepatocellular carcinoma were included in both studies.

Our ranking of infectious pathogens is relatively consistent with a pilot study conducted by the European Centre for Disease Prevention and Control (ECDC) to determine the burden of influenza, measles, HIV, tuberculosis, campylobacterosis, salmonellosis, and enterohaemorrhagic *E. coli* in Europe. [6] The seven infectious diseases were ranked in a similar order, except for influenza, which was ranked lower in the ECDC study because their primary analysis only considered laboratory-confirmed cases, which dramatically underestimates the burden. [6]

In contrast to previous studies reporting a slight dominance of the contribution of premature mortality over morbidity for infections, [2], [4] we found most of the burden was from premature mortality. This discrepancy relates to methodological differences in calculating ONBOIDS HALYs and GBD DALYs, with the use of more severe disability weights, discounting, age-weighting, and standard life expectancy all impacting the relative contribution of morbidity versus premature mortality. Despite uncertainty regarding which method should be considered the gold standard, it is reassuring that the rankings of these infectious diseases were similar.

The overall burden of infectious diseases was comparable in males and females, with marked differences for certain pathogens. Some differences have clear biological explanations, such as the differential burden of HPV (cervical cancer in females), and *E. coli* (anatomical differences resulting in more urinary tract infections in females). The difference for HIV is related to behaviours (i.e., anal intercourse among men who have sex with men and injection drug use, more prevalent among males). [36] For HBV and HCV, the differences may be due to a combination of biological and behavioural risk factors. [37], [38]

The syndrome-based results can guide prioritization of ‘horizontal’ prevention methods that are independent of the causative pathogen (e.g., smoking cessation and hand hygiene as methods of preventing pneumonia). Further work is needed to assess the benefits of possible interventions to prevent each of the syndromes and pathogens. Part of such an analysis could address the possibility of clustering of risk factors including social determinants of health.

The level of public concern and media attention for some pathogens is disproportionate to their actual disease burden. For example, substantial media attention has been directed to recent outbreaks of *Listeria monocytogenes* and West Nile virus in Ontario, [39], [40] but these pathogens accounted for only a small proportion (0.04% and 0.07%, respectively) of the total infectious disease burden. In contrast, top pathogens such as HCV, HBV, and *S. pneumoniae* receive little media coverage, yet are both burdensome and preventable. Since media reports often influence decision-makers, generating robust estimates of disease burden may improve decision-making.

With the exception of linkable healthcare utilization data, the data used in this study are readily available in most high-income settings. Studies like ONBOIDS could be conducted in many other jurisdictions with similar data sources by adapting the methods and parameter estimates used in ONBOIDS.

One major assumption of burden of disease studies is that the relationship between incidence and mortality remains constant over time. This may be less applicable to infectious diseases as incidence and severity frequently vary over time. We also did not consider the impact of co-infections (e.g., HIV and HCV) and other co-morbidities (e.g., diabetes and influenza virus). Important synergies exist between infectious diseases; notably, HIV can interact with other infectious diseases in terms of natural history and transmissibility. [41] While ONBOIDS is the most comprehensive examination of infectious diseases to our knowledge, the study was not exhaustive. We excluded certain pathogens (e.g., norovirus), syndromes (e.g., surgical site infections), and health states (e.g., amputations due to serious infection). The most significant exclusions may have been the oncogenic pathogens *Helicobacter pylori* and Epstein-Barr virus, given our finding that other oncogenic pathogens are among the most burdensome infectious agents. We also were unable to assess many milder infections that do not result in healthcare utilization, or take a societal perspective in assessing the impact of outbreaks (e.g., economic, psychological). Other important limitations include: the uncertain validity of the diagnostic codes for ascertaining infectious diseases; the assumption that etiologic agent distributions from studies of non-fatal outcomes also apply to fatal outcomes; reliance on a single underlying cause of death, which may have led to underestimation of the true burden of infectious diseases (e.g., deaths hastened or precipitated by infectious diseases would likely be attributed to pre-existing conditions); and the missed burden of undiagnosed infections. Finally, data were extracted from multiple sources of varying quality. Further details concerning the limitations of this work are described in the ONBOIDS technical report.

Despite these limitations, our study represents the most thorough examination of the population burden of infectious diseases to date. These results provide a crude ordering of infectious diseases that can be used to guide policy, planning, and research. Although data limitations preclude a greater level of precision and quantification of uncertainty, these results provide a sense of the relative importance of the infectious diseases studied. Less important than the precise position on the list is the fact that the top 20 agents capture such a large proportion of the total burden and that some recent high profile infections are absent.

The next step is to translate these findings into information that is tailored for local decision-making. The WHO GBD project has had significant impacts on global and regional agencies, but a provincial analysis such as ONBOIDS provides information closer to the level of government responsible for healthcare policy-setting and funding. However, these findings are likely generalizable to other high-income settings. In translating findings such as these into policy, decision-makers must appreciate the “herd-dynamic” aspects of infectious diseases, such that, for example, funding for highly successful vaccination programs is not diverted towards the high-impact diseases identified here. The communicable nature of vaccine-preventable diseases means that ongoing investment is necessary to *maintain* existing successes. Also, while novel interventions (e.g., new vaccines) are required to further reduce the burden of infectious diseases, much of the burden can already be reduced by improved implementation of existing interventions (e.g., hand hygiene, improved vaccine uptake, safe injection sites). Setting priorities requires knowledge of disease burden as well as critical evaluation of the feasibility, cost, and impact of available interventions, and knowledge translation for decision-makers. Future work should assess the economic and other broad societal burdens associated with infectious diseases.

## Supporting Information

Text S1 **HALY formulae.**
(DOCX)

Table S1 **Syndromes and episode lengths.**
(DOCX)

Table S2 **Parameters for estimating the disease burden due to** ***Salmonella*** **.**
(DOCX)

Table S3 **Parameters for estimating the disease burden due to human immunodeficiency virus (HIV).**
(DOCX)

Table S4 **Parameters for estimating the disease burden due to human papillomavirus (HPV)-related cancers.**
(DOCX)

Table S5 **Parameters for estimating the disease burden of various health states of human papillomavirus (HPV).**
(DOCX)

Table S6 **Parameters for estimating the disease burden due to** ***Streptococcus pneumoniae*.**
(DOCX)
