# Supplementary material for: The Impact of Infection on Population Health: Results of the Ontario Burden of Infectious Diseases Study
Source: PLoS One. 2012 Sep 4;7(9):e44103. doi: 10.1371/journal.pone.0044103 (PMC3433488; doi:10.1371/journal.pone.0044103)
Supplement: Text S1 — HALY formulae. (DOCX) [file pone.0044103.s001.docx]

**Supplementary Material**

**Text S1. HALY Formulae**

The health-adjusted life year (HALY) includes both death occurring before a pre-specified maximal life expectancy (i.e., years of life lost due to premature mortality [YLL]) and years of healthy life lost due to suboptimal states of health associated with disease (i.e., year-equivalents of reduced functioning due to disease [YERF]).

HALY = YLL + YERF

To calculate YLL, we multiplied the number of deaths (N) for a specific age (a) and sex (s) group due to a particular cause (c) by the standard loss function (L) (i.e., the remaining life expectancy for that age and sex stratum). The YLL for each age-sex group were summed to obtain the YLL for each infectious disease.

YLL*_c_* = Σ (N*_c,a,s_* * L*_a,s_*)

To calculate YERF, we multiplied the number of incident cases (I) of each associated health state (h) for a specific age (a) and sex (s) group by the average duration (D) of the health state (in years) and the severity weight (SW) derived for that health state. We summed the YERF, first for each age-sex group and then across health states to obtain the YERF for each disease.

YERF*_c_* = Σ (I*_c,h,a,s_* * D*_c,h_* * SW*_c,h_* )
