# Supplementary material for: The Impact of Infection on Population Health: Results of the Ontario Burden of Infectious Diseases Study
Source: PLoS One. 2012 Sep 4;7(9):e44103. doi: 10.1371/journal.pone.0044103 (PMC3433488; doi:10.1371/journal.pone.0044103)
Supplement: Table S1 — Syndromes and episode lengths. (DOCX) [file pone.0044103.s002.docx]

**Supplementary Material**

**Table S1. Syndromes and episode lengths**

| **Syndrome** | **Episode length*** |
| --- | --- |
| Acute bronchitis | 30 days |
| Bacterial meningitis | 3 years |
| Bronchiolitis | 30 days |
| Cellulitis | 30 days |
| Cervicitis | 30 days |
| Conjunctivitis | 15 days |
| Endocarditis | 60 days |
| Necrotizing fasciitis | 60 days |
| Orchitis/epididymitis | 30 days |
| Osteomyelitis | 60 days |
| Otitis media | 30 days |
| Pelvic inflammatory disease | 60 days |
| Pharyngitis | 30 days |
| Pneumonia | 30 days |
| Septic arthritis | 60 days |
| Septicaemia | 30 days |
| Upper respiratory tract infection | 15 days |
| Urethritis | 30 days |
| Urinary tract infection |  |
| Cystitis | 30 days |
| Pyelonephritis | 60 days |
| Acute prostatitis | 60 days |

*Episode lengths are the periods during which repeat healthcare utilization events for the same patient were considered to represent the same episode of infection.
