# Supplementary material for: The Impact of Infection on Population Health: Results of the Ontario Burden of Infectious Diseases Study
Source: PLoS One. 2012 Sep 4;7(9):e44103. doi: 10.1371/journal.pone.0044103 (PMC3433488; doi:10.1371/journal.pone.0044103)
Supplement: Table S2 — Parameters for estimating the disease burden due to Salmonella . (DOCX) [file pone.0044103.s003.docx]

**Supplementary Material**

**Table S2. Parameters for estimating the disease burden due to *Salmonella***

| **Health state** | **Percentage of *Salmonella* cases that progress to each health state** | | **Duration**  **(days)** | | **Severity weight** |
| --- | --- | --- | --- | --- | --- |
| Gastroenteritis – mild | 85[1] | 5.5[1] | | 0.023 | |
| Gastroenteritis – moderate | 15[1] | 10.5[1] | | 0.041 | |
| Gastroenteritis – severe | 2[1] | 16.0[1] | | 0.086 | |
| Septicaemia | 5[2] | 7.0[1] | | 0.652 | |

**References**

1. Kemmeren J, Mangen M, van Duynhoven Y, Havelaar A (2006) Priority setting of foodborne pathogens: Disease burden and costs of selected enteric pathogens. Netherlands: National Institute for Public Health and the Environment (RIVM). Available: <http://www.rivm.nl/bibliotheek/rapporten/330080001.pdf>. Accessed 6-20-2011.

2. Saphra I, Winter JW (1957) Clinical manifestations of salmonellosis in man; an evaluation of 7779 human infections identified at the New York Salmonella Center. N Engl J Med 256: 1128-1134.
