# Supplementary material for: The Impact of Infection on Population Health: Results of the Ontario Burden of Infectious Diseases Study
Source: PLoS One. 2012 Sep 4;7(9):e44103. doi: 10.1371/journal.pone.0044103 (PMC3433488; doi:10.1371/journal.pone.0044103)
Supplement: Table S3 — Parameters for estimating the disease burden due to human immunodeficiency virus (HIV). (DOCX) [file pone.0044103.s004.docx]

**Supplementary Material**

**Table S3. Parameters for estimating the disease burden due to human immunodeficiency virus (HIV)**

| **Health state** | | **Duration**  **(years)** | | **Severity weight** |
| --- | --- | --- | --- | --- |
| HIV | 20.00 | | 0.035 | |
| AIDS | 1.70 | | 0.247 | |
| AIDS – terminal phase | 0.08 | | 0.801 | |

AIDS=Acquired immunodeficiency syndrome
