# Supplementary material for: The Impact of Infection on Population Health: Results of the Ontario Burden of Infectious Diseases Study
Source: PLoS One. 2012 Sep 4;7(9):e44103. doi: 10.1371/journal.pone.0044103 (PMC3433488; doi:10.1371/journal.pone.0044103)
Supplement: Table S4 — Parameters for estimating the disease burden due to human papillomavirus (HPV)-related cancers. (DOCX) [file pone.0044103.s005.docx]

**Supplementary Material**

**Table S4. Parameters for estimating disease burden due to human papillomavirus (HPV)-related cancers**

| **Cancer site** | **Percentage attributed to HPV** | **Stage** | **Percentage at diagnosis** | **Severity weight for diagnosis*** | **Five-year survival** | **Surgery only** | **Radiation only** | **Surgery & radiation** | **Chemotherapy and radiation** | **Surgery, chemotherapy & ratiation** |
| --- | --- | --- | --- | --- | --- | --- | --- | --- | --- | --- |
| Cervix | 99.7[1] | I | 52 | 0.109 | 80[2] | 60[3] |  |  | 25 | 15 |
|  |  | II | 21 | 0.147 | 65 |  |  |  | 100 |  |
|  |  | III/IV | 27 | 0.191 | 30 |  | 10 |  | 90 |  |
| Vulva | 36–40[4] | I/II | 66 | 0.109 | 85 | 80[3] |  |  | 10 | 10 |
|  |  | III/IV | 34 | 0.191 | 25 | 35 |  |  | 50 | 15 |
| Vagina | 90[4] | All | 100 | 0.147 | 54[5] |  | 10[3] |  | 90 |  |
| Anal canal | 85[4] | I/II | 56 | 0.109 | 80[6] |  | 5[6] | 95 |  |  |
|  |  | III/IV | 44 | 0.147 | 55 |  | 5 | 85 |  | 10 |
| Penis | 50[4] | All | 100 | 0.109 | 85 | 100 |  |  |  |  |
| Oropharynx | 33–72[4] | I/II | 18 | 0.109 | 85[7,8] |  | 100[9] |  |  |  |
|  |  | III/IV | 82 | 0.147 | 70 |  | 40 |  | 60 |  |

Note: for ranges, the midpoint was used in estimates

*SW for Dx was dependent on prognosis (i.e., five-year survival)

**References**

1. Munoz N, Castellsague X, de Gonzalez AB, Gissmann L (2006) Chapter 1: HPV in the etiology of human cancer. Vaccine 24 Suppl 3: S3-1-S310.

2. Fauci, AS, Braunwald, E, Kasper, DL, Hauser, S, Longo, D, Jameson, J, and Loscalzo, J (2008) Harrison's Principles of Internal Medicine. New York: McGraw-Hill Professional.

3. Murphy J, Kwong JC (2010) Personal communication with Dr. Joan Murphy, University Hospital Network, Toronto, Canada.

4. Giuliano AR, Tortolero-Luna G, Ferrer E, Burchell AN, de Sanjose S, Kjaer SK, Munoz N, Schiffman M, Bosch FX (2008) Epidemiology of human papillomavirus infection in men, cancers other than cervical and benign conditions. Vaccine 26 Suppl 10: K17-K28.

5. Kosary CL (1994) FIGO stage, histology, histologic grade, age and race as prognostic factors in determining survival for cancers of the female gynecological system: an analysis of 1973-87 SEER cases of cancers of the endometrium, cervix, ovary, vulva, and vagina. Semin Surg Oncol 10: 31-46.

6. Cummings B, Kwong JC (2010) Personal communication with Dr. Bernard Cummings, University Health Network, Toronto, Canada.

7. Galati LT, Myers EN, Johnson JT (2000) Primary surgery as treatment for early squamous cell carcinoma of the tonsil. Head Neck 22: 294-296.

8. Hull MC, Morris CG, Tannehill SP, Werning JW, Amdur RJ, Hinerman RW, Villaret DB, Mendenhall WM (2003) Definitive radiotherapy alone or combined with a planned neck dissection for squamous cell carcinoma of the pharyngeal wall. Cancer 98: 2224-2231.

9. Poon I, Kwong JC (2010) Personal communication with Dr. Ian Poon, Sunnybrook Health Sciences Centre, Toronto, Canada.
