# Supplementary material for: The Impact of Infection on Population Health: Results of the Ontario Burden of Infectious Diseases Study
Source: PLoS One. 2012 Sep 4;7(9):e44103. doi: 10.1371/journal.pone.0044103 (PMC3433488; doi:10.1371/journal.pone.0044103)
Supplement: Table S5 — Parameters for estimating the disease burden of various health states of human papillomavirus (HPV). (DOCX) [file pone.0044103.s006.docx]

**Supplementary Material**

**Table S5. Parameters for estimating the disease burden of various health states of human papillomavirus *(*HPV)**

| **Health state** | **Severity weight** | **Duration** |
| --- | --- | --- |
| Surgery | 0.268 | 4 weeks |
| Radiation | 0.219 | 6 weeks |
| Chemotherapy | 0.250 | 2 weeks |
| Remission | 0.035 | Lifetime |
| Palliative care | 0.516 | 5 months |
| Terminal care | 0.821 | 1 month |
