# Supplementary material for: The Impact of Infection on Population Health: Results of the Ontario Burden of Infectious Diseases Study
Source: PLoS One. 2012 Sep 4;7(9):e44103. doi: 10.1371/journal.pone.0044103 (PMC3433488; doi:10.1371/journal.pone.0044103)
Supplement: Table S6 — Parameters for estimating the disease burden due to Streptococcus pneumoniae. (DOCX) [file pone.0044103.s007.docx]

**Supplementary Material**

**Table S6. Parameters for estimating disease burden due to *Streptococcus pneumoniae***

| **Health state** | **Percentage of syndrome attributable to *S. pneumoniae*** | **Duration** | **Severity weight** | **Episode length*** |
| --- | --- | --- | --- | --- |
| Bacterial meningitis (BM) | 32 (0–4 years)  45 (5–14 years)  51 (≥15years)[1,2] | 2 weeks[3] | 0.652 | 3 years |
| Sequelae of BM** |  |  |  |  |
| Seizures | 24[2] | Permanent | 0.039 | N/A |
| Motor deficits | 65[2] | Permanent | 0.062 | N/A |
| Deafness | 9[4] | Permanent | 0.071 | N/A |
| Septicaemia | 4.8[5] | 1 week[6] | 0.652 | 30 days |
| Pneumonia | 30[7] | 2 weeks | 0.136 | 30 days |
| Septic arthritis | 1[8] | 19 days[9] | 0.108 | 60 days |
| Acute bronchitis | 20[10] | 2 weeks[11] | 0.086 | 30 days |
| Otitis media | 26[12] | 4.5 days[13] | 0.052 | 30 days |
| Conjunctivitis | 12[14] | 1 week[15] | 0.023 | 15 days |

* Episode length denotes the amount of time that must have elapsed between occurrences of the infection in the health care utilization data to be considered separate events in a single individual.

** Percentage of cases that progress to health state

**References**

1. Nigrovic LE, Kuppermann N, Malley R (2008) Children with bacterial meningitis presenting to the emergency department during the pneumococcal conjugate vaccine era. Acad Emerg Med 15: 522-528.

2. van de Beek D, de Gans J, Spanjaard L, Weisfelt M, Reitsma JB, Vermeulen M (2004) Clinical features and prognostic factors in adults with bacterial meningitis. N Engl J Med 351: 1849-1859.

3. Fauci, AS, Braunwald, E, Kasper, DL, Hauser, S, Longo, D, Jameson, J, and Loscalzo, J (2008) Harrison's Principles of Internal Medicine. New York: McGraw-Hill Professional.

4. Kutz JW, Simon LM, Chennupati SK, Giannoni CM, Manolidis S (2006) Clinical predictors for hearing loss in children with bacterial meningitis. Arch Otolaryngol Head Neck Surg 132: 941-945.

5. Byl B, Clevenbergh P, Jacobs F, Struelens MJ, Zech F, Kentos A, Thys JP (1999) Impact of infectious diseases specialists and microbiological data on the appropriateness of antimicrobial therapy for bacteremia. Clin Infect Dis 29: 60-66.

6. Kemmeren J, Mangen M, van Duynhoven Y, Havelaar A (2006) Priority setting of foodborne pathogens: Disease burden and costs of selected enteric pathogens. Netherlands: National Institute for Public Health and the Environment (RIVM). Available: <http://www.rivm.nl/bibliotheek/rapporten/330080001.pdf>. Accessed 6-20-2011.

7. File TM (2003) Community-acquired pneumonia. Lancet 362: 1991-2001.

8. Goldenberg DL (1998) Septic arthritis. Lancet 351: 197-202.

9. Imboden, JB, Hellmann, DB, and Stone, JH (2004) Current Rheumatology: Diagnosis & Treatment. New York: McGraw-Hill Medical.

10. Creer DD, Dilworth JP, Gillespie SH, Johnston AR, Johnston SL, Ling C, Patel S, Sanderson G, Wallace PG, McHugh TD (2006) Aetiological role of viral and bacterial infections in acute adult lower respiratory tract infection (LRTI) in primary care. Thorax 61: 75-79.

11. Wenzel RP, Fowler AA, III (2006) Clinical practice. Acute bronchitis. N Engl J Med 355: 2125-2130.

12. Guven M, Bulut Y, Sezer T, Aladag I, Eyibilen A, Etikan I (2006) Bacterial etiology of acute otitis media and clinical efficacy of amoxicillin-clavulanate versus azithromycin. Int J Pediatr Otorhinolaryngol 70: 915-923.

13. Rothman R, Owens T, Simel DL (2003) Does this child have acute otitis media? JAMA 290: 1633-1640.

14. Gigliotti F, Williams WT, Hayden FG, Hendley JO, Benjamin J, Dickens M, Gleason C, Perriello VA, Wood J (1981) Etiology of acute conjunctivitis in children. J Pediatr 98: 531-536.

15. Foulks G, Gordon J, Kowalski R (2000) Bacterial Infections of the Conjunctiva and Cornea. In: Albert D, Jakobiec FA, editors. Principles and Practice of Ophthalmology. Philadelphia: WB Saunders Company.
